# Social learning and culture in birds: emerging patterns and relevance to conservation

**DOI:** 10.1098/rstb.2024.0128

**Published:** 2025-05-01

**Authors:** Lucy Aplin, Ross Crates, Andrea Flack, Peter McGregor

**Affiliations:** ^1^ Department of Evolutionary Biology and Environmental Studies, University of Zurich, Zurich 8057, Switzerland; ^2^ Research School of Biology, Australian National University, Canberra, Australian Capital Territory 2600, Australia; ^3^ Cognitive and Cultural Ecology Research Group, Max Planck Institute of Animal Behavior, Radolfzell 78315, Germany; ^4^ Fenner School of Environment and Society, Australian National University, Canberra, Australian Capital Territory 2600, Australia; ^5^ Collective Migration Group, Max Planck Institute of Animal Behavior, Konstanz 78464, Germany; ^6^ Eco-Ethology Research Unit, Instituto Universitário, ISPA, 1149-041 Lisbon, Portugal

**Keywords:** animal culture, conservation, birds, social learning, migration, bird song

## Abstract

There is now abundant evidence for a role of social learning and culture in shaping behaviour in a range of avian species across multiple contexts, from migration routes in geese and foraging behaviour in crows, to passerine song. Recent emerging evidence has further linked culture to fitness outcomes in some birds, highlighting its potential importance for conservation. Here, we first summarize the state of knowledge on social learning and culture in birds, focusing on the best-studied contexts of migration, foraging, predation and song. We identify extensive knowledge gaps for some taxa but argue that existing evidence suggests that: (i) social learning and culture are taxonomically clustered and that (ii) reliance on social learning in one behavioural domain does not predict reliance across others. Together, we use this to build a predictive framework to aid conservationists in species-specific decision-making under imperfect knowledge. Second, we review evidence for a link between culture and conservation in birds. We argue that understanding which behaviours birds are likely to learn socially can help refine conservation strategies, improving the trajectories of threatened populations. Last, we present practical steps for how consideration of culture can be integrated into conservation actions including reintroductions, translocations and captive breeding programmes.

This article is part of the theme issue ‘Animal culture: conservation in a changing world’.

## 1. Introduction

Aves (hereafter birds) encompasses over 11 000 species across 36 orders, ranging from enormous ratites to tiny hummingbirds, to aquatic penguins. Yet despite this diversity, the majority of birds exhibit parental care [1] and are social, with social systems ranging from monogamous territorial pairs to cooperative family groups and vast aggregations [2]. A few taxonomic groups of birds such as the Corvidae and Psittaciformes even rival great apes in their social complexity [3]. For example, species like common ravens (*Corvus corax*) exhibit extended parental care of multiple months and adult lifespans of several decades [4] coupled with a variety of socio-cognitively complex behaviours including alliance formation, differentiated social networks and extended social memory [5]. This is reflected in their brain neuronal countings, with high densities of neurons concentrated in the telencephalon (forebrain) and absolute neuron counts comparable to primates [6].

Altogether, this combination of life-history traits would suggest that the large majority of birds are capable of social learning and possess the basic building blocks for animal culture. Indeed, song learning in passerines is one of the best-studied forms of animal culture, with abundant evidence from over a hundred years of research that many passerines socially learn songs from older models during development [7] and can exhibit geographical variation in song dialects [8]. To note, here we follow accepted convention and the other papers in this special issue by defining *social information use* as the use of information from others to inform decision-making [9], *social learning* as the acquisition of skills, knowledge or behaviour from interaction or observation of other individuals and their products [10] and *animal culture* as socially learned behavioural variants that are shared in groups and retained over time [11].

While work on song has provided the majority of studies on animal culture in birds, there is increasing evidence for social learning or culture in a variety of behavioural domains, including other forms of vocal communication [12], diet and foraging behaviour [13,14], tool use [15], threat recognition [16], nest building [17] and migration [18]. Much of this evidence has derived from relatively short-term experiments in model species such as zebra finches (*Taeniopygia guttata*) [19], great tits (*Parus major*) [20], chickens (*Gallus gallus domesticus*) [21], New Caledonian crows (*Corvus moneduloides*) [22,23] and budgerigars (*Melopsittacus undulatus*) [24]. In some cases, there is additional evidence that this social learning leads to cultural inheritance of behaviour, where skills or knowledge are transmitted across generations [25,26]. In a smaller subset of cases, social learning has also been shown to lead to cultures [13,15,17]. Collectively, this work is beginning to reveal how social learning influences behaviour in a range of contexts, the complex interactions between social learning, experience and genes in shaping phenotypes, and the circumstances that lead to formation of cultures [27].

Theory suggests that individuals should continue to refine their behavioural repertoire by selectively retaining the most beneficial behaviour variants and then honing those behavioural variants with practice [28,29]. It therefore follows that animal cultures will tend to be locally adaptive [30], and the acquisition or loss of cultural traits will have potential fitness consequences [31]. If cultural traits have fitness consequences, then it further follows that retention of the capacity for culture in animal populations may be important for conservation, and the loss or erosion of existing cultures may also have associated conservation outcomes [32]. This theoretical link between animal culture and conservation was recently formalized in two reviews by Brakes *et al*. [33,34]. While most empirical evidence to date has come from primates [35], there is a growing body of research in birds showing a link between population declines or fragmentation with changes in song cultures or migration behaviour.

The evidence for the capacity for social learning, culture and cultural evolution in birds has been reviewed in previous work [16,36–38], and we do not propose to provide a comprehensive coverage here. Rather, we have three aims, all of which arise out of the avian working group at previous meetings of the UN Environment Programme (UNEP) Convention of Migratory Species (CMS) Expert Working Group on Animal Culture and Social Complexity. First, we review the taxonomic distribution of evidence for social learning and culture across the four best-studied behavioural contexts of predator responses, foraging, migration and vocal communication and identify the potential links to fitness and vital rates in each case [33]. Second, we use this to build a predictive framework for the presence and form of cultures across the avian phylogeny, with the aim of assisting decision-making in the face of the knowledge gaps that exist for many taxa. Finally, we highlight the practical measures that conservationists can take to integrate understanding of culture in their decision-making.

## 2. The evidence for social learning and culture in birds

As stated above, there is widespread evidence for social learning in birds [16,27,36,37], and we do not aim to provide a comprehensive review. Instead, we focus on summarizing that evidence in four behavioural domains: threat responses, foraging, migration and vocal communication. These represent contexts where most research has been concentrated and where we consider that a loss of, or change in, these behaviours are likely to have conservation implications.

### (a) Social learning about danger

Many birds exhibit mobbing behaviour, where individuals group together to harass potential threats. This gives ample opportunities for the social transmission of knowledge about what predators look like [39] , the level of threat they pose [40,41] and the alarm calls themselves [42]. This was first experimentally demonstrated in captive blackbirds (*Turdus merula*), where naive individuals learned to mob a novel object when observing or hearing a conspecific mob it [43]. More recently, cultural transmission of predator recognition has been shown in the wild. For example, in one study, American crows (*Corvus brachyrhynchos*) were captured by people wearing distinctive masks. Mobbing responses by crows to people wearing these masks were then socially transmitted to naive crows and juveniles, with mobbing responses persisting in the population for at least 5 years [39,44]. Birds are also capable of socially learning about the threat of cuckoos through mobbing. For example, naive superb fairywrens (*Malurus cyaneus*) and reed warblers (*Acrocephalus scirpaceus*) initially show little response to cuckoos, but after observing conspecifics mob a cuckoo, will begin to exhibit mobbing [45,46]. Finally, it is also possible for individuals to socially learn specific alarm calls. In one notable example in fairywrens, simply pairing a known conspecific alarm call with a novel heterospecific alarm call was sufficient to elicit learning that persisted over time, suggesting acoustic–acoustic associative learning mechanisms [47].

Despite the abundant evidence for social learning of predator responses in birds, there is little evidence that such responses lead to cultural variation across populations. In many cases, it is instead clear that social learning operates to hone existing cognitive biases. For instance, in the example above, reed warblers socially learned to mob a cuckoo but did not respond after observing a neighbour mob a harmless parrot [45]. Similarly, blackbirds more easily learned to fear a novel bird than a plastic bottle, although fear responses could still be socially learned in either case [48]. Furthermore, there should be a strong selective pressure to successfully recognize predators and identify their threat level, with threat levels fairly consistent across groups and environments. It, therefore, seems likely that even without underlying cognitive biases, groups should rapidly converge on similar behavioural responses. While there is more potential for cultural variation to arise in the alarm calls themselves, here the evidence also suggests that acoustic properties of many avian alarm calls are conserved, with this genetic architecture likely providing a selective advantage by eliciting faster learning [49,50].

Whether predator responses are entirely socially learned or whether social learning operates to hone conserved cognitive or sensory biases, it seems self-evident that such learning will have fitness consequences, as fast and accurate recognition of predators or of alarm calls will directly impact survival [16]. In contrast, knowledge about predators can often be rapidly lost in captive or predator-free populations, such as those found on islands [51], with potentially dramatic outcomes for conservation [52]. The mechanisms leading to predator naivety in birds are multifaceted [53]. However, if predator responses are socially influenced, this gives an opportunity to borrow experimental paradigms from social learning research [54] to either train knowledge of invasive predators into wild populations or to efficiently retrain captive populations pre-release [55,56].

**Figure 1.**
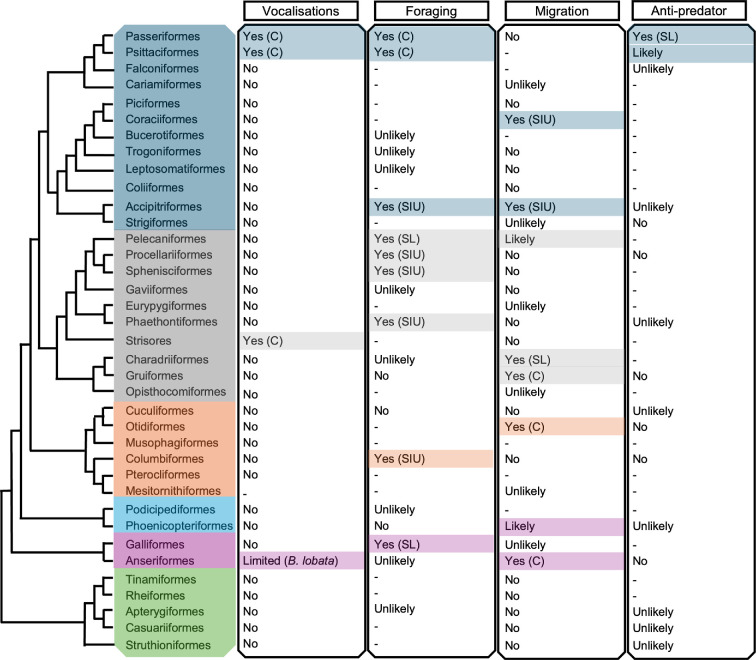
A predictive framework for the occurrence of cultural traits across avian orders. Cultural traits are separated into four groups: vocalisations, foraging, migration and anti-predator responses. Taxa are classified as ‘Yes’ (direct evidence for multiple species in that order), ‘Limited’ (direct evidence only for one taxonomic group), ‘Likely’ (no direct evidence, but likely based on life history), ‘Unlikely’(no direct evidence, not likely based on life history), ‘No’ (no evidence, despite studies that have investigated this question), and ‘-’ (unknown, with no scientific research). In the case of a ‘Yes’ or ‘Limited’ classification, the evidence is further categorized into evidence for culture (C), social learning (SL) or social information use (SIU).

To date, almost all empirical research on social learning about danger has been conducted in passerines, including corvids (e.g. *Coloeus monedula*), starlings and mynahs (e.g. *Sturnus vulgaris* and *Acridotheres tristis*), Eurasian blackbirds and honeyeaters (e.g. *Manorina melanocephala*) (see [16] for review). Notably, almost all these studies have been conducted on adult birds, providing indirect evidence that predator responses can be socially learned throughout life. In contrast, outside of Passeriformes, there is extremely limited evidence (figure 1). Predator recognition was socially influenced in gulls (order Charadriiformes), although individuals were also able to directly observe a predated conspecific, and so also had opportunity to gather personal information about the threat [57]. Similarly, in pre-release predator training in houbara bustards (*Chlamydotis undulata,* order Otididae), pairing conspecific alarm calls with a predator only lead to sustained predator responses if the individuals experienced a live fox that posed an active threat to the focal individual [58]. In both cases, therefore, individuals had to personally experience the predation threat to learn, although their responses may have been modulated by the presence of conspecifics. On the current evidence, this would suggest that cultural transmission of knowledge about predators and other threats may be largely restricted to passerines. However, it is important to note that clearly there is an immense gap in knowledge for non-passerine birds, and generalizations are impossible without further research.

### (b) Learning about diet and foraging behaviour

When compared with the extensive study of foraging cultures in other groups like primates [59–61]), we know surprisingly little about the occurrence, form or function of foraging cultures in birds. However, what studies we do have suggest that use of social learning to acquire dietary knowledge is likely to be widespread in birds [62], and the acquisition of foraging techniques may often be facilitated by parents, leading to cultural inheritance of the various aspects of the foraging niche [26,36]. For example, in a classic experiment on Eurasian oystercatchers (*Haematopus ostralegus*), cross-fostered chicks acquired the ‘hammering’ or ‘stabbing’ mussel opening technique of their foster parents [63]. Oystercatchers often also exhibit individual dietary specializations, and it has been further speculated that these may be culturally inherited, with this leading to within-population cultural polymorphisms [46]. Similarly, in two sympatric passerines, blue tits (*Cyanistes caeruleus*) and great tits (*P. major*), interspecific cross-fostering experiments revealed that individuals tend to adopt the diet and foraging microhabitat of their foster species [29]. Recent experiments on these species suggest individuals may also learn ‘*what not’* to eat, with information on avoidance of potentially dangerous food rapidly transmitted through social networks [62].

Unlike in taxa such as primates or cetaceans [61,64–66] where researchers have relied on the ethnographic method to identify variation in long-lasting foraging cultures between populations [59], the evidence for foraging cultures in birds has mostly been derived from studies of the spread of innovations [20]. For example, in Aplin *et al*. [20], the authors seeded knowledge of how to solve a foraging puzzle into wild populations of great tits and observed the behaviour spreading across social networks to establish as a multi-generational foraging tradition. This work demonstrated that innovations on the part of a very few individuals are sufficient, in some species, to lead to the emergence of foraging cultures, supporting previous observations of innovative foraging in this species [67,68].

Naturally occurring innovations are also widely reported in birds, with innovativeness correlated with species and individual-level traits such as brain size, generalism, behaviour plasticity and neophobia [69]. In a few cases, these innovations have been observed to spread to form local traditions. For example, in one recent observational study on sulphur-crested cockatoos (*Cacatua galerita*), the geographical spread of an urban foraging innovation (bin-lid opening) was tracked over several years [13]. In this case, there was additional evidence that spatially distant areas were beginning to develop distinct subcultures in bin-opening techniques, giving a rare insight into how between-population variation in foraging cultures might first emerge [13,70]. Such innovations can also include the adoption of novel foods, such as the emergent local tradition for eating hibernating bats observed in one population of insectivorous great tits in Hungary [67].

The evidence for long-established foraging cultures in birds is rarer, with probably the example coming from tool use in New Caledonian crows [71]. In this species, individuals undertake multiple steps to construct tools for extracting wood-boring grubs, with mastery of this behaviour facilitated by extended parental care and access to the discarded tools of adults [22,72,73]. Tool types exhibit cultural variation across the species range [74,75], and the complexity of tool forms also varies spatially in a way that some argue is indicative of cumulative cultural evolution [15]. Finally, these wild observations have been coupled with a series of captive studies examining learning mechanisms across development [23,76], making tool use in New Caledonian crows perhaps the best-understood avian foraging culture outside of the context of changing environments.

Overall, the taxonomic reach of foraging cultures in birds appears to be broad, with evidence from passerines [15,20], parrots [13,77] and shorebirds [63]. Evidence for social learning of foraging behaviour or dietary knowledge is even more widespread, including in more basal bird orders like Galliformes (but see [21,78]). Finally, while not specifically discussed here, social information use is widespread across most social species, with evidence for the use of conspecifics as a local enhancement to find food in diverse taxa from swallows [79] to seabirds [80]. In addition to this taxonomic breadth, while cultural inheritance of foraging behaviour from parents and adults is likely to be important, there is no evidence that social learning of foraging behaviour is otherwise restricted to a sensitive development period [81]. While speculative, we would argue that this suggests that learning of diet or foraging behaviour is unlikely to rely on specialized cognition or neural architecture but is more likely to involve broadly prevalent mechanisms including local and stimulus enhancement [82,83]. Rather, it seems likely that foraging cultures will be most likely to occur in species that rely on extractive foraging, where learned foraging techniques will be most useful [81].

To date, there has been very little direct evidence linking foraging cultures to fitness in birds. However, foraging cultures are generally thought to represent adaptations to local resource conditions [84,85], and such a link has been demonstrated in other taxa (e.g. primates [35]). Indeed, as discussed above, foraging cultures in birds are most often described in the context of behavioural responses to changing environments, with local traditions emerging that range from eating cream [68] or opening bins [13] in suburbia to predating hibernating bats in unusually harsh winters [67]. In such cases, while fitness is not directly measured, the adaptive benefit of the behaviour appears to be clear. Furthermore, while the link between foraging cultures and conservation is largely unexplored in birds, the emergence of culture in changing environments further suggests a direct link with population resilience. If so, maintaining the capacity for innovations to arise and spread in populations may be a vital component of conservation planning in the Anthropocene [30].

### (c) Social learning of migration

It is clearly established that the migratory behaviour of many bird species is largely genetically encoded and shaped by natural selection [86]. Multiple studies have shown that inexperienced young birds from various species depend on an inherent directional programme when performing their first migration [87]. However, in recent years, it has become evident that in a subset of bird species, migrations are not solely determined by genetics but are also shaped by individual and social learning and enhanced through cultural evolution across generations [18,38]. Furthermore, it has been shown that social transmission of migratory knowledge can outperform individual learning and facilitate learning in critical developmental periods [38].

However, although it is often stated that social learning may be a crucial process by which migration knowledge transmits between generations [18,38], strong empirical evidence for social learning of migratory decision-making comes almost exclusively from a few long-lived taxa that migrate in family units (e.g. Anatidae: geese, Gruidae: cranes and Laridae: terns). Due to the difficulty of tracking multiple generations in the same flock over long time periods, studies seldom go beyond revealing the potential for social information transfer by observing route efficiency in relation to flock composition [88,89]. Yet, there is evidence that social learning from experienced birds can facilitate long-term increases in migration accuracy in whooping cranes, *Grus americana* [90]. More specifically, this study capitalizes on a unique dataset of reintroduced whooping cranes that originate from a captive breeding programme. Knowing the relatedness of all individuals, it reveals that the age of the oldest bird in the flock, rather than genetic relatedness, predicts migratory performance.

In other systems, information transfer from parents to offspring may lead to cultural inheritance, as recently shown in Caspian terns (*Hydroprogne caspia*). By migrating together with their fathers, juvenile terns not only learn their migration routes, but they also increase their survival rates. In terns, this form of socially learned migration does not, however, lead to group-level cultures, as there is still high variation in routes within the population [91]. In contrast, cultural inheritance can lead to migratory cultures in geese, which also travel in family groups. For example, recent studies have shown that cultural inheritance is a key driver of novel migration behaviour in pink-footed geese, *Anser brachyrhynchus* [92], and barnacle geese, *Branta leucopsis* [93,94] . In these two cases, cultural transmission also facilitates adaptation to changing environments. For pink-footed geese, research documented the rapid formation of a new migration route and breeding population in Russia, facilitated by warming temperatures and cultural transmission of migration behaviour []. Barnacle geese have expanded their range northward in response to climate change and population growth, with individual experiences influencing this shift [93,94]. Thus, migrating in flocks with mixed ages and levels of experience can lead to the emergence of novel migratory patterns, like shorter routes or new stopover grounds, that spread in the population through social learning [92,95,96]. This can buffer against environmental change and generally increase flexibility in socially learning migrants.

However, it is important to note that the specific transmission modes associated with migratory cultures can also influence how strongly migratory birds are impacted by global change. While the potential for horizontal spread of knowledge about new routes or stopover grounds can promote adaptive flexibility, as shown above in geese [92–94], a strong vertical across-generation transmission of migration behaviour might also act to maintain established traditions and reduce the ability of populations to respond to environmental change [97]. In addition, juveniles of populations that already suffered decline may experience reduced social learning opportunities, reducing survival rates for these young birds and threatening the population even further [98,99]. Migrants also regularly move through unfamiliar regions and face unpredictable environmental conditions or predation threats, all of which will impact fitness. Migrating in social groups may weaken initial selection pressures on suboptimal routes and timing [100,101]. This, therefore, links social group sizes with success, and inversely, suggests potential negative fitness outcomes linked to population declines. Thus, maintaining population demography may be vital for conservation of social learning migratory species.

To date, evidence for migratory cultures in when, where and how to migrate appears to be largely concentrated in a few taxonomic groups, including cranes, waterfowl and shorebirds. However, social migration in single or multi-species aggregations is much more taxonomically widespread, giving the potential for a broader role for social information use. Beyond safety in numbers, migrating in large flocks of mixed age may provide information on migratory direction, suitability of flyways and habitats or environmental conditions [102,103]. For example, for large aggregations of nocturnally migrating passerines, social interactions through vocalisation may improve navigational decision-making during long-distance flights [104,105]. In addition, when migrants rely on environmental support from wind, social information can improve the detection of beneficial conditions through collective sensing and lead to more energetically efficient flight trajectories [106]. Similarly, although stopovers can have various functions [107], social interactions likely impact the decisions of when and where to stop [108,109]. Even more, relying on social information during stopovers may affect foraging success and predation risks at these unknown sites [110]. We need further research pairing long-term ecological observations with experimental manipulations to explore whether and, if so, how socially induced decisions transmit across generations to create cross-generational persistence.

### (d) Social learning and vocal communication

Almost all bird species vocalise and do so through specializations of the syrinx and associated muscle and neural control systems [111]. These vocalisations function in social interactions such as breeding, feeding and avoiding predators, all of which have obvious fitness implications. More complex and longer vocalisations are termed song and are distinguished from calls [93]. Here, we focus on song learning (vocal production learning) for singing in the context of social interactions, predominantly breeding and resource defence [112]. While social learning of calls is likely to be common (e.g. social and alarm calls, see below), evidence remains relatively scarce (but see, e.g. [113,114]).

Most information on social learning and culture in birds comes from a long history of laboratory and field studies on vocal production song learning, with a focus on male song and singing behaviour of a few temperate zone passerines. Early laboratory studies (e.g. [115]) have been considered evidence for a general song learning pattern: details learned from singing adults are added to an inherited species-specific song template during a sensitive period (when the learner is a nestling and/or a fledgling) after which song is crystallized and does not change substantially thereafter [116]. Studies of song learning in the wild have reported more varied learning patterns [7], including species that continue to learn songs throughout life and species that make concerted changes within populations [117] and cumulative cultural evolution [118]. The role of social interaction in song sparrow (*Melospiza melodia*) song development has been established by laboratory and field studies and illustrates some likely general consequences for function and development (reviewed by [119]), that are driven by the fitness benefits of song sharing with territorial neighbours (e.g. [120]), likely because song matching and complexity are often integral to mate choice ([121], but see [122]). Similarly, singing the current version of the population- and time-specific song dialect likely has fitness benefits that can result in concerted, population-wide change in song variants [117].

The key role of social interactions in song learning in the wild can also lead to adverse consequences for fitness. In conservation-relevant contexts of declining and fragmented populations, song learning patterns can include individuals learning songs from a different species (often a more common congeneric, e.g. [32]). The potency of social interaction to override the presumed inherited song template was demonstrated in laboratory experiments where live tutors replaced taped tutoring, leading to a species of North American sparrow (white-crowned sparrows, *Zonotrichia leucophyrs*) singing the song of a species of African finch [123] .

This variety of learning patterns in the wild underscores the likely importance of adaptive behavioural differences between species [124] and of social interactions. There are very few studies that integrate song development with song function, meaning there is little information on how or why birds choose to learn the songs they sing from the songs around them, and the fitness consequences of such choices (but see, [118]). A better understanding of the selective advantages of learning and singing particular song variants would make clearer the potential links with conservation, including better inference of effective population size from the pattern of vocal variation and the effect of augmenting or translocating threatened populations by release of captive bred individuals [31].

Taxonomically, song is a defining characteristic of the Oscines (or Passeri), a sub-order of the Passeriformes, and the evidence for song cultures overwhelmingly comes from this group. However, the common division of Passeriformes into songbirds that learn songs (Oscines) and Suboscines that do not learn songs is arguably more of a hindrance than a help when using taxonomic information to identify the presence and form of song cultures. This is partly because other avian orders show vocal learning, notably parrots (Psittaciformes) and hummingbirds (Trochilidae), as does at least one genus of Suboscines, the bellbirds (*Procnias*) [125]. It is also partly because there is a growing recognition that vocal production learning is a continuum ranging from convergence in calls to advanced learning underlying song production [126]. For example, Moran *et al*. [127] investigated vocal learning in New Zealand wrens (sub-order Acanthisitti), a group that shares a common ancestor with parrots and Oscines. They found evidence for limited vocal learning in the vocal convergence of feeding calls of rifleman (*Acanthisitta chloris*) whereby the call features of interacting individuals come to resemble one another even though they are not closely related. This and other studies have led to a re-evaluation of vocal production learning beyond the Oscines, with some evidence for limited learning found in six other orders [128]. Therefore, while the present weight of evidence suggests that vocal production learning shows a strong phylogenetic signal, and this has guided our presented framework (figure 1), we lack full understanding of the evolutionary predictors of this trait. It would seem prudent to assume that the taxonomic range of species exhibiting vocal production learning will expand with future research [128].

## 3. Recommendations for integrating understanding of avian cultures into conservation action

The need to better integrate our understanding of animal culture into applied conservation actions is increasingly being acknowledged [33,34]. In birds, evidence for socially learned and culturally maintained behaviours is not congruent with their threat status [129], and there is limited evidence for how socially learned behaviours might compromise or aid conservation efforts. Gaining such knowledge is often not a priority for conservation programmes, especially when balanced against urgent needs such as addressing the direct drivers of population decline and ensuring the longer term viability of *ex situ* populations [130].

Yet even with the limited current knowledge base, multiple opportunities exist to better integrate existing knowledge on social learning and animal culture into applied management actions (box 1, see [31,32,95,131–135]), and there is real potential for conservation gains in doing so [136]. Therefore, when time and resources are not available to gain knowledge on the occurrence, form and patterning of potential culture, we would give two initial recommendations. First, a quick assessment tool kit could be used to ascertain whether the behaviour of interest is socially learned, as discussed by Whiten & Rutz [137] in this issue. Second, a more immediate and pragmatic approach is to use phylogeny and life histories to predict from current information whether conservation of a species of concern is likely to be influenced by social learning or culture in one or more behavioural domains. We have attempted to go some way to assisting this by providing a summary of the distribution of current knowledge on social learning and culture across the avian phylogeny (figure 1). However, given how widespread culture and social learning are in birds, and its ecological importance, we would recommend implementing this with a precautionary approach [138, 139].

Box 1. **Integrating culture into avian conservation actions involving captive breeding and reintroduction.**

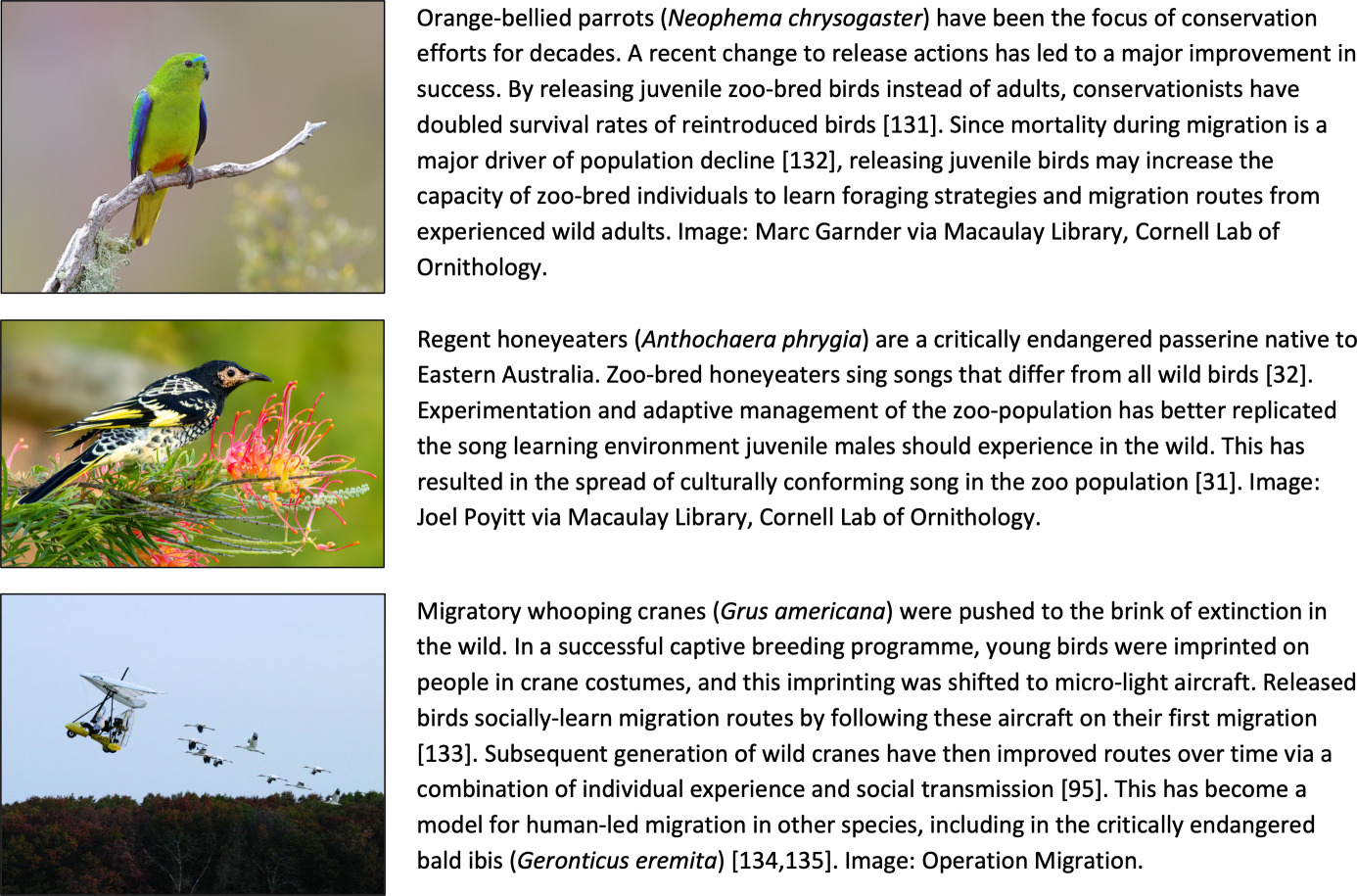

Two examples illustrate this two-part approach. First, multiple species of migratory birds from taxa including waterfowl, storks, cranes, pelicans, raptors, bee-eaters and shorebirds are known to exhibit social influences on migration, suggesting this may be widespread in these groups [18,38,106]. This suggests that maintaining population density will be a priority when aiming to retain healthy migratory patterns. A subset of these species that are long-lived and live in family groups (e.g. geese, pelicans and swans) are also known to exhibit cultural inheritance of specific routes [90,93,140] . This suggests that in the case of these life histories, retaining age structure in populations is an additional priority. However, our knowledge of the distribution of vocal learning across birds suggests that vocal cultures are unlikely to occur in the aforementioned taxa (figure 1), and thus providing opportunities to learn vocalisations will not be a priority for investigation or conservation action. Second, and at the other extreme, to our knowledge there is no direct evidence for social learning of any behaviours in kiwis (Apterygiformes). Yet we do know that kiwi species are resident, largely solitary foragers, basal to the first known vocal learning taxa and evolved without most predators [141]. Migratory and vocal cultures are thus very likely to be absent, and social learning of foraging or anti-predator behaviour is unlikely. Conservation action integrating social learning should therefore not need to be integrated into current approaches [142], though a rapid assessment may still be useful in the latter two cases to exclude the possibility [137].

### (a) Which cultures to conserve?

It is important to note that we do not necessarily propose that conservation action aims to retain all existing cultures that are ascertained to be present. It may indeed be desirable in cases where populations have honed particular behaviours over multiple generations, for example, in the case of specific migration routes [90] or complex tool use [15]. But in most other cases, retaining the capacity for culture will be the greater priority. As §2 outlines, there is widespread evidence in birds of a capacity to reinvent or innovate cultures in healthy populations [13,20]. For example, bird songs often exhibit change over space and time in dialect. To conserve such constantly changing variants in the wild would be difficult and potentially counterproductive. However, conserving the capacity for behavioural variation in song that underpins mate choice and successful breeding clearly has high conservation value. Similarly for foraging behaviours, maintaining capacity to innovate new behaviours is vital; indeed, this capacity is likely to be an important source of behavioural responses to rapid environmental change [143,144].

When retaining the capacity for culture is the goal, we recommend this be achieved through aiming to maintain three essential elements: *capacity to re/invent*, *capacity to transmit* and *capacity to retain*. Although what exactly this will involve will vary between species and contexts, we would suggest that it usually includes focusing on maintaining: (i) social density within populations or groups, (ii) connectivity between populations or groups and (iii) connectivity between generations through age structure.

### (b) Conserving cultures in captivity

Captive breeding for reintroduction has long been known to often lead to the loss of behaviour [145]. While the extent to which this loss compromises the success of avian reintroduction efforts remains little studied, it is becoming increasingly clear that changes (invariably reductions) in the extent and nature of social interactions individuals experience in captivity can lead to substantial differences in socially learned behaviours compared with wild counterparts [145]. This is exemplified by differences in song culture between wild and zoo-bred regent honeyeaters (*Anthochaera phrygia*; box 1), where captive populations developed a highly simplified song, likely resulting in poor reproductive success of these individuals after release [31].

While the specific mechanisms underlying the acquisition and retention of behaviours are often poorly characterized, we can recommend two practical steps that will conserve cultural behaviours in captive populations, even without knowledge of these mechanisms. First is to consider social interactions when designing the physical layout of breeding and holding facilities, as the scope for social interactions (and hence transmission of behaviours) will be affected by the size, design, number and orientation of aviaries. Second, when recruiting individuals to act as founders of captive populations, older adults will most often be the best option, as adults will have had the opportunity to learn behaviours in the wild which they can potentially transfer to others in captivity. Furthermore, the number and ratio of wild founders will influence the capacity of cultures to be maintained in captivity in the longer term [139,146]. Similarly, increasing the rate at which individuals are exchanged between wild and captive populations could be an important way of helping maintain cultures in captivity.

Finally, if socially learned behaviours cannot be maintained passively in captive populations via these steps, it may be possible to actively sustain them through tutoring programmes (see [52]). Such programmes are increasingly being used to restore key behaviours in a range of avian taxa, including vocalisations [136] and antipredator behaviours [56]. Tutoring animals in captivity offers the further potential to help seed adaptive behaviours in the wild, such as conditioned aversion to exotic species.

### (c) Conserving cultures during reintroductions and translocations

Integrating understanding of animal culture into conservation will have major implications for reintroduction strategies. First, many behaviours such as migration routes and vocalisations are typically learned in early life [90,147], so releasing juveniles or a ‘younger than average’ cohort may offer those individuals the best chance to learn behaviours from wild conspecifics. Second, individuals should be released into populations where and when wild birds are present, facilitating social information transfer from wild to zoo-bred individuals. For many mobile bird species including migrants and nomads, such a strategy would require a degree of flexibility in reintroduction approaches. For more sedentary species, the key consideration would be timing reintroductions to coincide with periods in which societies are more fluid, such as the post-breeding period, when captive-bred birds have the greatest opportunity to assimilate into wild flocks [148]. Finally, the release process itself may also provide an opportunity to seed adaptive behaviours back into groups; an approach that has been most often applied to migration (box 1; [133,135]). In one notable example in northern bald ibis (*Geronticus eremita*), post-release training of migratory behaviour has been used as an opportunity to establish an entirely new migratory route that will be more adaptive under future climate change [134].

It is also vital to consider the presence and form of animal culture when planning translocation strategies. First and foremost, if a species exhibits socially learned behaviours, it is also more likely to flexibly respond to translocations. For example, previous work has demonstrated that bird species that learn migratory behaviour have more capacity to flexibly shift migratory routes or even cease migration altogether in novel and changing environments [93,95,96]. Second, active cultural rescue may also be attempted through translocation processes. For example, Alberts lyrebirds (*Menura alberti*) living in smaller rainforest fragments have depleted cultural mimetic song repertoires relative to conspecifics occurring in larger fragments (and therefore larger societies [149]). Similarly to genetic rescue, translocation of individuals from larger to smaller subpopulations could help increase the mimetic repertoire size of individuals occurring in smaller subpopulations, potentially enhancing their long-term viability. Yet it is vitally important to consider the form and function of cultural behaviours when assessing whether translocations are likely to have positive local effects. For instance, if individuals exhibit a different vocal dialect, and dialects are important for mate choice or social interactions, this may jeopardize their capacity to integrate into local populations.

## 4. Conclusion

There is evidence for a role of social learning and cultural inheritance in shaping various behaviours in a large range of avian taxa. Yet this still represents a tiny proportion of the approximately 11 000 avian species, and more research is needed on many neglected taxa (figure 1). However, in those taxa that have been studied, patterns are increasingly emerging for an uneven phylogenetic distribution of the presence of social learning and culture in different behavioural domains. This is best studied for vocal learning, which is currently considered to be restricted to six distinct lineages (but see, [128]). Overall, this allows for the beginning of a predictive framework to guide both future research and management practices.

In addition to evidence for the occurrence of social learning and culture, evidence is growing in many bird species for a cyclic interaction between culture and conservation, with population declines leading to cultural drift, simplification or loss and this loss of culture linking to negative fitness outcomes. Animal cultures are one of many considerations for conservation programmes that are invariably limited by time, funding and knowledge gaps. In such cases, conserving the capacity for culture may be sufficient and align with general goals of maintaining healthy population sizes and connectivity. However, in many cases, by explicitly considering which behaviours birds learn socially, there may be opportunities to make simple changes to conservation strategies that could substantially improve the trajectories of threatened species’ populations.

## Data Availability

This article has no additional data.
